# Grief trajectories and long-term health effects in bereaved relatives: a prospective, population-based cohort study with ten-year follow-up

**DOI:** 10.3389/fpubh.2025.1619730

**Published:** 2025-07-25

**Authors:** Mette Kjærgaard Nielsen, Kaj Sparle Christensen, Mette Asbjoern Neergaard, Pernille Envold Bidstrup, Mai-Britt Guldin

**Affiliations:** ^1^Department of Mental Health, Research Unit for General Practice, Aarhus, Denmark; ^2^Department of Public Health, Aarhus University, Aarhus, Denmark; ^3^Section for Specialist Palliative Care, Department of Oncology, Aarhus University Hospital, Aarhus, Denmark; ^4^Department of Clinical Medicine, Aarhus University, Aarhus, Denmark; ^5^Psychological Aspects of Cancer, Danish Cancer Institute, Copenhagen, Denmark

**Keywords:** grief, bereavement, relatives, general practice (GP), health care use, mortality, medication use, primary care

## Abstract

**Background:**

Bereavement may affect the health of relatives, causing increased use of health care services and increased mortality shortly after the patient's death. However, the long-term consequences for those with a high level of grief symptoms remain largely unexplored. We aimed to investigate associations between grief symptom trajectories and four long-term health outcomes among relatives bereaved by natural death: contacts to general practice and mental health services, use of psychotropic prescription medication, and mortality, over a period of 3–10 years post-bereavement.

**Method:**

We assessed grief symptoms using the Prolonged Grief-13 scale in a cohort of 1,735 bereaved relatives at three different time points (prior to bereavement, 6 months after bereavement, and 3 years after bereavement) and identified five main grief trajectories. The trajectory with persistent low levels of grief symptoms in relatives [n=670 (45%)] was called the low grief trajectory (LGT) and was used as reference. The high grief trajectory (HGT) consisted of 107 (6%) relatives with persistent high grief symptom levels. We investigated associations between grief trajectories and (1) contacts to general practitioner (GP) including out-of-hours using negative binomial regression analysis, (2) contacts to mental health services (GP talk therapy, private-practice psychologist or psychiatrist), (3) use of psychotropic medications (antidepressants, anxiolytics and sedatives) using logistic regression analysis, and (4) mortality using Cox regression analysis. The follow-up period started at 3 years after bereavement and long-term outcome were further followed until 10 years after the patient's death.

**Results:**

Relatives in the HGT had a significantly higher yearly incidence of GP contacts until seven years after bereavement compared to the LGT. The HGT was associated with higher use of mental health services [OR = 2.86 (95% CI 1.58; 5.19)], antidepressants [OR = 5.63 (95% CI 3.52; 9.01)], sedatives and anxiolytics [OR = 2.60 (95% CI 1.63; 4.14)], and excess mortality [HR = 1.88 (95% CI 1.1; 3.2)] compared to the LGT.

**Conclusion:**

This study shows that patients with high and sustained grief symptoms have an increased healthcare use up to 10 years after loss. Future research should assess whether current health care services sufficiently meet the prolonged needs of these relatives.

## Introduction

The death of a close relative due to severe illness is a life-changing event for most people. According to the recently developed integrated process model of loss and grief, the death of a significant other is a multidimensional experience causing suffering and may contain both physical, emotional, cognitive, social, and spiritual dimensions (1). Bereavement may affect the mental and physical health, including development of depression and heart failure (2). Hence, the reactions to loss cause increased use of health care services and higher mortality of relatives immediately after the death of a close relative (3). Register-based studies have shown that bereaved relatives often have a higher use of psychologist sessions after bereavement (4), a higher risk of being hospitalized (4, 5), and being prescribed more psychotropic medication, such as antidepressants and sedatives, compared to their non-bereaved peers (4). However, we lack knowledge of whether the most vulnerable relatives use more health care services and whether there is an impact on their mortality in a long-term perspective.

A large amount of research has been conducted on Prolonged Grief Disorder in recent years and diagnostic criteria have been described (6). However, grief reactions that not necessarily meet these criteria may describe common reactions to loss and may affect the bereaved relative and their long-term reactions to bereavement. A few bereavement studies have included grief symptoms beyond the first year after the death of a close relative and have focused on the prevalence of grief symptoms (7–11). Between 2 and 20% of bereaved relatives experienced prolonged grief symptoms during the 5 years following bereavement (7–10). In a population-based sample, 1/3 of the population reported that they had experienced severe grief due to bereavement measured by a single question (11). Low socio-economic status, low self-reported health, higher symptoms of depression and anxiety and higher use of health care services were associated with more severe grief (11). This is in line with prior studies concerning predictors of adverse outcome (12, 13). The long-term impact on mental and physical health after bereavement was found to be affected, especially in those with limited social contact (14) and may impact the daily life of relatives for years.

Mortality in connection with bereavement has been investigated in register-based studies (15–17). Increased mortality risk has been found after sudden or traumatic bereavement, loss of a child, and prior mental health conditions such as depression (3, 15–17). Yet, reduced mortality has also been found in bereaved relatives (18, 19), and the authors draw attention to benefits of caregiving as it may buffer the distress of relatives (the stress-buffering effect) (19).

Thus, bereavement has been shown to have a long-term impact on bereaved relatives (3, 15, 20). However, follow-up studies of long-term effects of bereavement have not included data on grief symptoms and short-term psychological reactions. As the reactions of bereaved relatives are heterogenous (21), we need more knowledge on whether patterns of grief and psychological reactions may predict the long-term wellbeing and psychological consequences.

In a prior study, we examined the development of relative's grief symptoms based on the Prolonged Grief-13 Scale (22) by conducting grief trajectories from short time before to three years after the patient's death in a cohort of 1,735 relatives (23, 24). We identified five distinct grief trajectories that described common reactions to illness and loss. Two of the five trajectories showed minimal differences in symptom level at the three time points of measurements. The *high grief trajectory* (HGT) constituted a small group of 107 persons (6%) who had a consisting of high levels of persistent grief symptoms, whereas 670 persons (38%) had a *low grief trajectory* (LGT) of persistent low levels of grief symptoms (24). The remaining three common grief trajectories were between these extremes and had fluctuating symptom levels. In all, 310 (18%) had a high/decreasing grief trajectory (HDGT) and 526 (29%) had a moderate/decreasing grief trajectory (MDGT), and both of these trajectories had high/moderate symptom levels before the patient's death that decreased after death. The last grief trajectory constituted 122 (9%) who had a late grief trajectory (LaGT) with low levels of grief symptoms before death with a peak at 6 months after the patient's death (23, 24). At 6 months before the death of the patient, we found that the HGT group had more frequent contacts to the GP, more GP talk therapy, and more prescriptions of antidepressants and sedatives compared to the LGT group (24).

In the present study, we aimed to investigate associations between these grief trajectories and the long-term use of primary healthcare, prescription medication and mental health services, as well as mortality from 3 years up to 10-years after bereavement.

We hypothesized that individuals with high grief symptom levels, especially those in the HGT group, would have a higher use of health care services and higher use of psychotropic medications. The HGT group was vulnerable in prior studies regarding reactions to illness and bereavement and we hypothesized that this group would have a higher mortality compared to the LGT group.

## Materials and methods

### Study design and setting

The study is based on a prospective, population-based cohort established in Denmark in 2012 and followed to 2022. The Danish health care system is tax-funded. Services are free of charge for residents, and GPs serve as gate-keepers to secondary care, including palliative care and psychiatric services (22). However, 40% of the costs of psychotherapy by a psychologist is self-paid after referral from a GP (23). Seven GP talk therapy sessions are free of charge per year, and GPs attending peer supervision may provide 20-min sessions focusing on mental health issues (24).

### Study population

**Figure 1:**
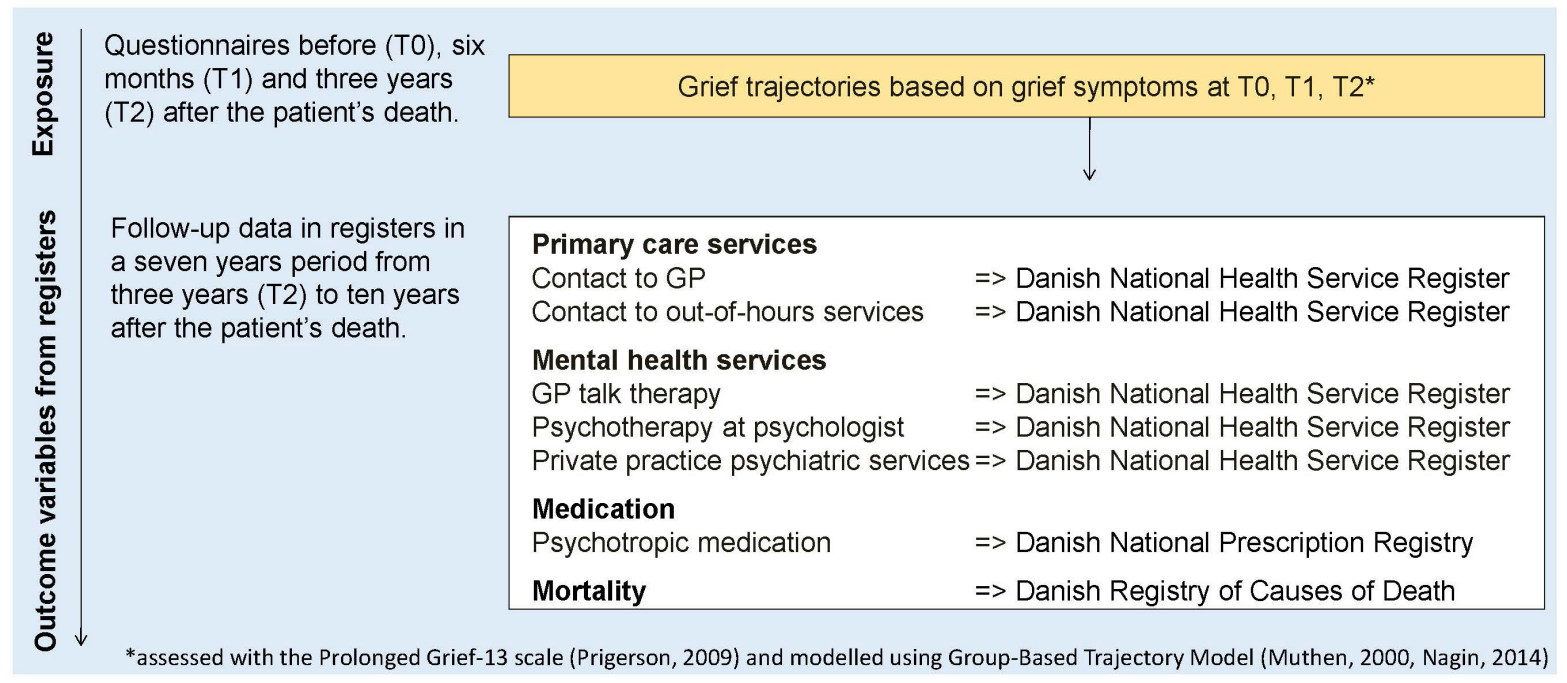
Timeline of exposure (grief trajectories) and register-based outcome variables.

In 2012, we obtained register-based information on all patients receiving drug reimbursement due to terminal illness, i.e. patients with a life-expectancy of only a few weeks or months (25). Weekly we sent letters to patients newly registered with drug reimbursement and asked for the patient's closest relative to complete the questionnaire (26). Enrolled relatives completed a questionnaire at the time of inclusion (T0), at 6 months after bereavement (T1), and at 3 years after bereavement (T2), and the outcome variables were followed in individual time periods for up to seven years after T2 (Figure 1). The study period ended December 31, 2022. Information regarding the relation to the patient (partner, adult child, other) was collected in the baseline questionnaire at T0.

### Grief trajectory exposures

The Prolonged Grief-13 (PG-13) scale (22) was used to measure grief symptoms at T0, T1, and T2. At baseline (T0), we used an adapted version of the scale (26) including “loss” changed to “illness” to correspond to the time prior to the patient's death in line with prior studies (27).

We have previously identified five specific grief trajectories based on the PG-13 measures at T0, T1, T2 using a semi-parametric group-based trajectory model (GBTM) for repeated measurements (28, 29). Details of the method have been described elsewhere (23, 24).

### Healthcare service, prescription medication and mortality outcomes

A unique personal registration number is granted to all Danish citizens. This number allows for linkage between individual-level records in Danish registers and the collected questionnaire data, which was processed at Statistics Denmark.

#### Contacts to general practice

GP contacts included total daytime contacts (face-to-face, prevention, talk therapy, phone, e-mail, and home visit), daytime face-to-face consultations, and out-of-hours (OoH) initial contacts (phone and video contacts) obtained from the Danish National Health Service Register (30).

#### Psychotropic medication

The use of psychotropic medication was measured as registered prescriptions on antidepressants (N06A), sedatives/hypnotics (N05C), or anxiolytics (N05B) according to the Anatomical Therapeutic Chemical (ATC) Classification System obtained from The Danish National Prescription Registry (31).

#### Mental health services

Mental health services included GP talk therapy (no, yes), psychotherapy sessions with a private-practice psychologist (no, yes) or private-practice psychiatrist (no, yes) after referral from a GP. The categories (GP talk therapy, psychologist and psychiatrist) were merged due to low numbers in the categories (30). The data was retrieved from the Danish National Health Service Register (30).

#### Mortality

We identified bereaved relatives who had died during follow-up. Data on dates of death was retrieved from the Danish Registry of Causes of Deaths (32).

### Covariates in adjusted analyses

Information on the age of the relative, gender, educational level according to the UNESCO international standard of classification [low ( ≤ 10 years), intermediate (>10 and ≤ 15 years), high (>15 years)] (33). The Danish National Patient Registry (34) provided information of diagnoses registered in connection with a contact to a hospital, which allowed us to adjust for the presence of at least one of the somatic diseases in the Charlson Comorbidity Index (CCI) (35).

### Statistical analysis

For all the analyses, we described the association between the five grief trajectories and outcome measures with those in the LGT as the reference group. Relatives contributed with at risk time up until date of death, date of emigration or December 31, 2022, whichever came first.

To examine the incidence rate ratios (IRRs) for GP contacts (daytime face-to-face and out-of-hours) according to grief trajectories (LGT as refence) we applied negative binomial regression analyses to account for overdispersion. Repeated yearly measurements were addressed using cluster robust variance estimation (36) and follow-up time was used as the offset. Cluster robust variance estimation was used to account for the repeated yearly measurements within each relative.

To examine the odds ratios (ORs) for the dichotomous outcomes mental health contacts and medicine use according to grief trajectories (LGT as reference) we applied logistic regression models. The dichotomous outcomes of mental health contacts and medicine redemptions were analyzed using logistic regression models with follow-up time used as the offset.

Finally, to examine the hazard ratios (HRs) for death according to grief trajectories (LGT as refence) we applied Cox proportional hazards models with age chosen as the underlying time scale. The proportional hazards assumption was assessed graphically using log-minus-log plots and no apparent violation was observed.

The negative binomial regression models yielded incidence rate ratios (IRRs) of yearly GP contacts, the logistic regression models yielded odds ratios (ORs) of medication use and mental health services within the seven-year follow-up period, and the Cox proportional regression model yielded hazard ratios (HRs) of mortality. All regression models were adjusted for the a-priori chosen covariates of age, gender, personal relation to deceased, education and CCI. All estimates were presented with 95% confidence intervals (CIs) and ratio estimates were considered statistically significant if 1 was not included in the CI. All analyses were done using Stata 18 (StataCorp, Texas, USA).

## Results

### Study population

The study population of 1,735 persons included predominantly females (71%). In all, 1,138 were partners (66%), 476 (27%) were adult children and 121 (7%) had another relation to the patient. The mean age was 62 years, 449 (26%) had a low education and 17% had one or more comorbid diseases (CCI≥1) (Table 1).

**Table 1:** Characteristics of the study cohort.

**Characteristics of participants**	**Participants (*n* = 1,735)**
Age, mean (CI^*^)	62.0 (61.5; 62.6)
**Gender**	
Male, *n* (%)	508 (29.3)
Female, *n* (%)	1,227 (70.7)
**Relation**	
Partner, *n* (%)	1,138 (65.6)
Children, *n* (%)	476 (27.4)
Other, *n* (%)	121 (7.0)
**Educational level**	
Low, *n* (%)	449 (25.9)
Intermediate, *n* (%)	828 (47.7)
High, *n* (%)	458 (26.4)
**Comorbidity at baseline** ^**^	
No (CCI = 0), *n* (%)	1,326 (82.7)
Yes (CCI ≥ 1), *n* (%)	278 (17.3)

^*^CI, Confidence Interval.

^**^CCI, Charlson Comorbidity Index.

### GP contacts during daytime and out-of-hours and associations with grief trajectories

**Figure 2:**
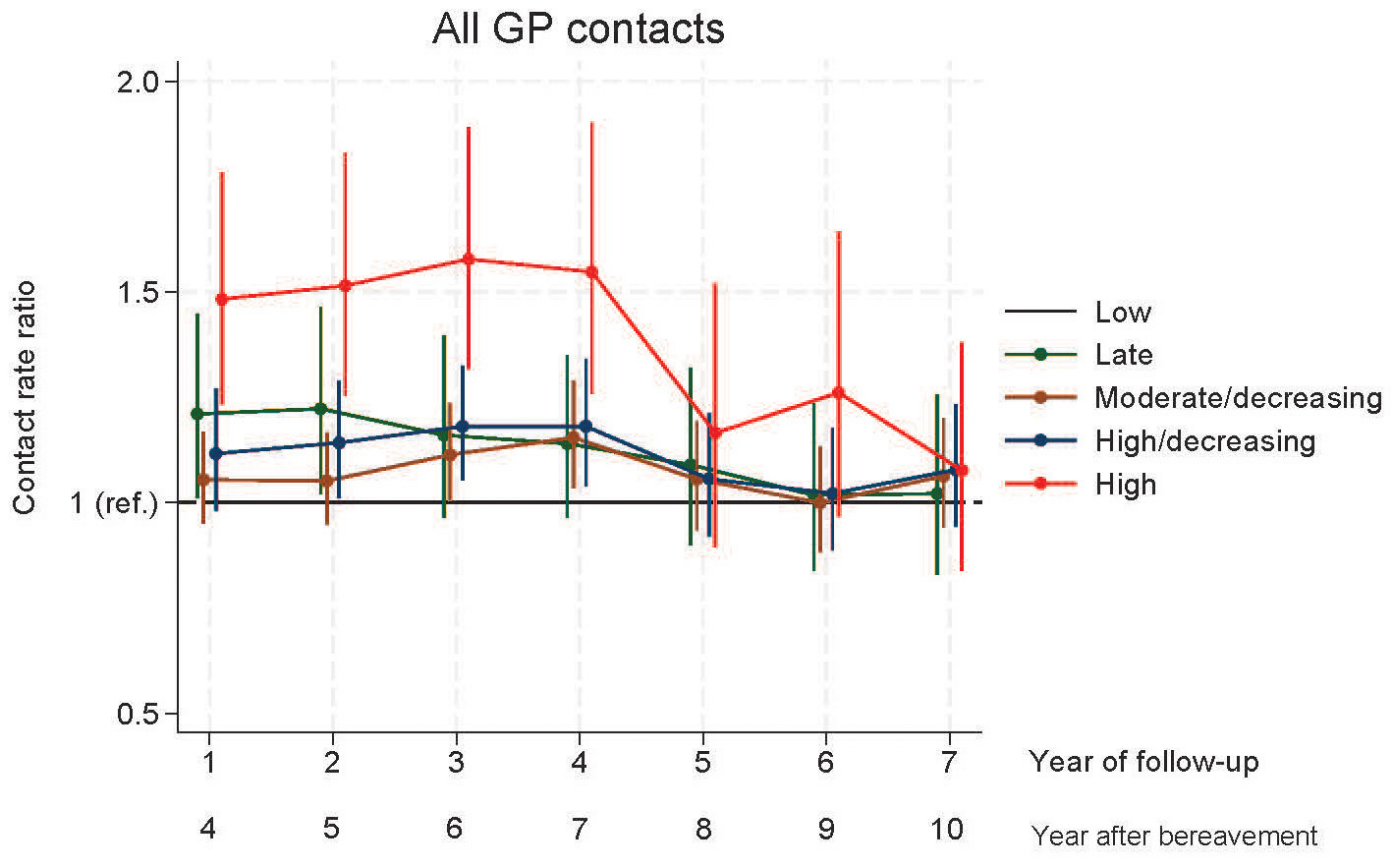
Contacts to general practice during daytime.

**Figure 3:**
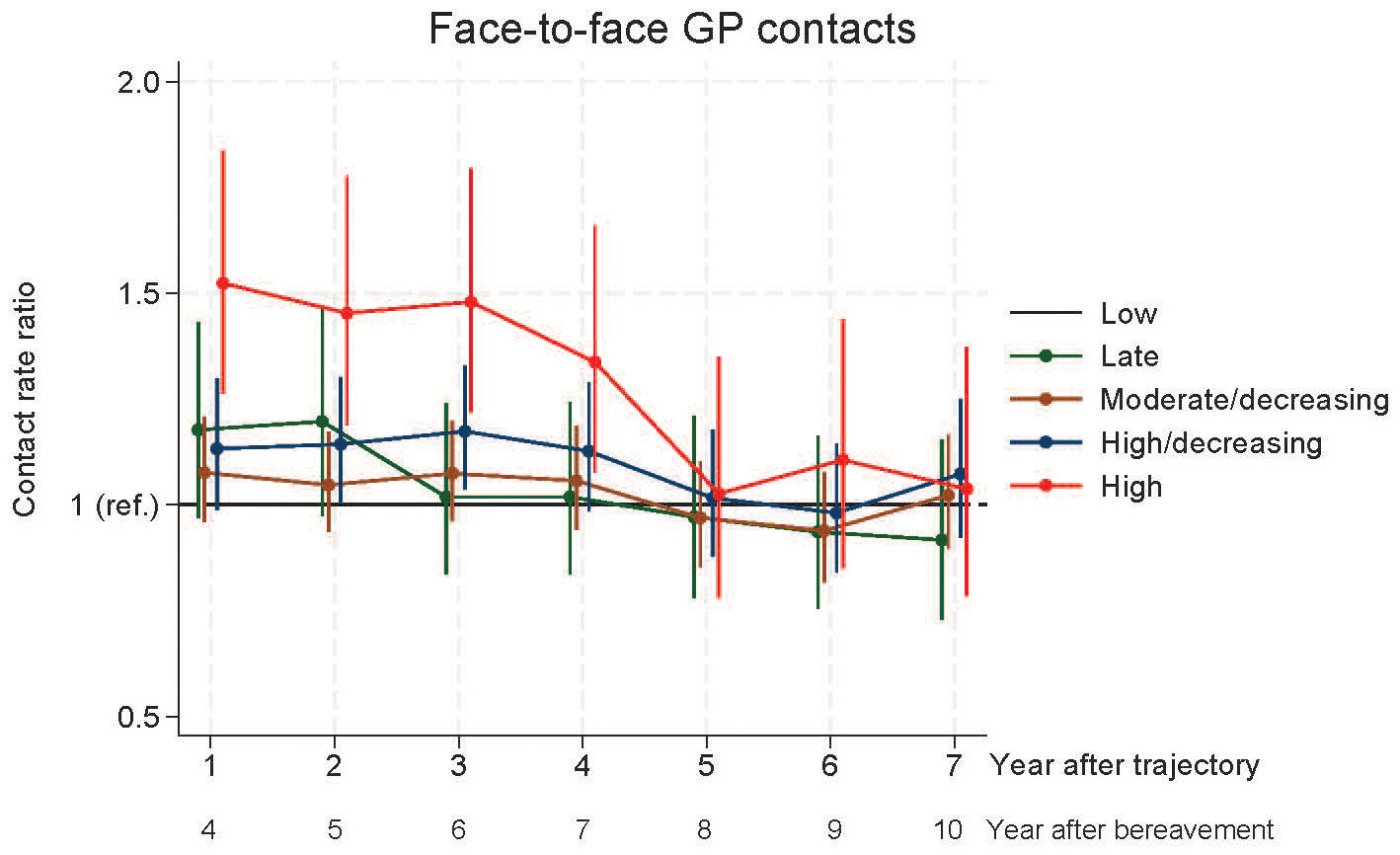
Face-to-face contacts to general practice.

Relatives in other GT groups than the reference group (LGT) had more GP contacts during the first years of follow-up and the difference seemed to level out toward 10 years of follow-up (Figures 2, 3). Relatives in the LaGT had more overall contacts 4 and 5 years after bereavement, but no statistically significant excess face-to-face contacts compared to relatives in the LGT.

Relatives in the HDGT had significantly more total contacts in year five to seven and more face-to-face contacts in year six after bereavement, whereas those in the HGT had significantly more GP daytime contacts (face-to-face contact and total contacts) in year four to seven after bereavement compared to the LGT (Figures 2, 3). From year eight to ten after bereavement, no significant difference in contact pattern was found between any of the grief trajectories.

**Figure 4:**
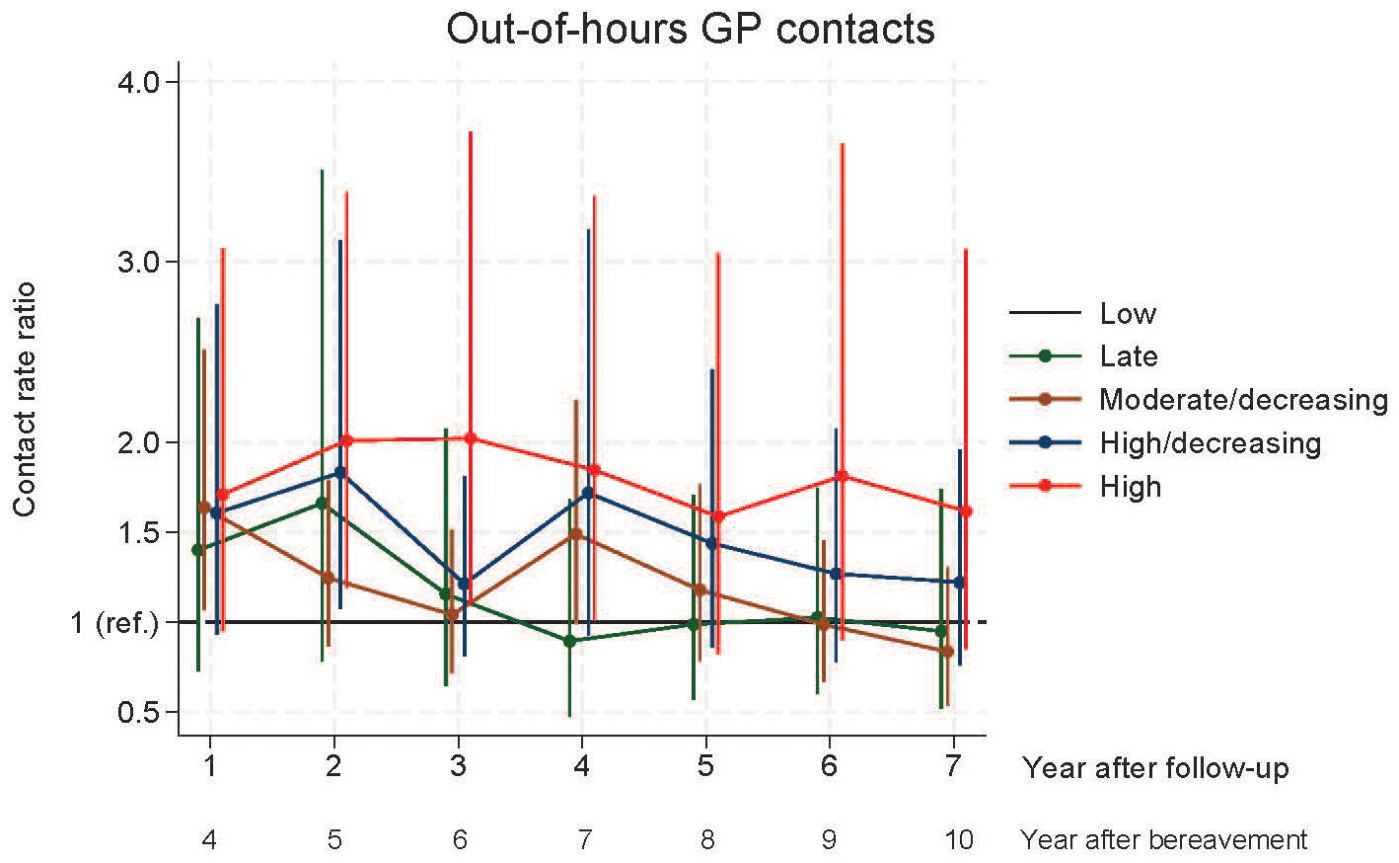
Contacts to general practice out-of-hours.

The pattern of initial contact with out-of-hours services showed that in year four after bereavement, the MDGT had significantly more contacts than the LGT, in year five the HDGT had more contacts and in year five and six, the HGT had significantly more contacts than the LGT (Figure 4).

### Mental health services and psychotropic medication

In the adjusted logistic regression analysis, the HGT was associated with more mental health services [OR = 2.86 (95% CI: 1.58;5.19)], use of antidepressants [OR = 5.63 (95%CI: 3.52;9.01)] and anxiolytics and sedatives [OR = 2.60 (95%CI: 1.63;4.14)] compared to the LGT (Table 2).

**Table 2:** Associations between grief trajectories and use of mental health care and medication.

***N* = 1,735**	**Number of events (*n*)**	**Follow up years**	**Adjusted OR^a^**	**95% CI**
**Contact to mental health services** ^bc^				
Low grief trajectory (*n* = 670)	71	4,536	1	-
Late grief trajectory (*n* = 122)	14	798	1.37	0.72; 2.6
Moderate/decreasing grief trajectory (*n* = 526)	60	3,510	1.18	0.81;1.74
High/decreasing grief trajectory (*n* = 310)	41	2,072	1.47	0.94; 2.30
High grief trajectory (*n* = 107)	20	676	2.86	1.58;5.19
**Psychotropic medication** ^b^				
**Antidepressants**				
Low grief trajectory (*n* = 670)	92	4,536	1	-
Late grief trajectory (*n* = 122)	35	798	2.46	1.53;3.97
Moderate/decreasing grief trajectory (*n* = 526)	77	3,510	1.04	0.74; 1.46
High/decreasing grief trajectory (*n* = 310)	74	2,072	1.87	1.29; 2.70
High grief trajectory (*n* = 107)	52	676	5.63	3.52; 9.01
**Anxiolytics and sedatives** ^b^				
Low grief trajectory (*n* = 670)	123	4,536	1	-
Late grief trajectory (*n* = 122)	33	798	1.59	0.99; 2.54
Moderate/decreasing grief trajectory (*n* = 526)	95	3,510	0.94	0.69;1.28
High/decreasing grief trajectory (*n* = 310)	91	2,072	1.77	1.26;2.48
High grief trajectory (*n* = 107)	41	676	2.60	1.63; 4.14

^a^Age, gender, personal relation, education, Charlson Comorbidity Index.

^b^According to an adjusted^a^ logistic regression model with the low grief trajectory as reference.

^c^GP talk therapy and/or sessions at private-practice psychologist or psychiatrist.

Use of antidepressants was also higher in the LaGT [OR = 2.46 (95% CI: 1.53;3.97)] and HDGT [OR = 1.87 (95%CI: 1.29;2.70)] and use of anxiolytics and sedatives was higher in the HDGT [OR = 1.77 (95%CI: 1.26;2.48)] compared to the LGT (Table 2).

### Mortality

In total, 186 (10.7%) died during 3–10 years after the patient's death, and the HGT was associated with mortality [HR = 1.88 (95% CI: 1.11; 3.21)] compared to the LGT (Table 3).

**Table 3:** Associations between grief trajectories and mortality.

***N* = 1,735**	**Number of deaths**	**% of trajectory group**	**Follow up years**	**Adjusted HR^a^**	**95% CI**
**Mortality** ^b^					
**Total**	**186**	**10.7**			
Low grief trajectory (*n* = 670)	49	7.3	4,536	1	-
Late grief trajectory (*n* = 122)	19	15.6	798	1.25	0.70; 2.20
Moderate/decreasing grief trajectory (*n* = 526)	54	10.3	3,510	1.20	0.80;1.80
High/decreasing grief trajectory (*n* = 310)	41	13.2	2,072	1.51	0.98; 2.33
High grief trajectory (*n* = 107)	23	21.5	676	1.88	1.11;3.21

^a^Age, gender, personal relation, education, Charlson Comorbidity Index.

^b^According to an adjusted^a^ Cox regression model with the low grief trajectory as reference.

## Discussion

Compared to relatives with a persistent low level of grief symptoms, we found that the grief trajectory groups with higher levels of symptoms had more contact to GP, higher use of mental health care and medication and excess mortality. This was significant for relatives in the HGTs of persistent grief symptoms for all outcome, including contact to GP in the time period until seven years after bereavement. Furthermore, those in the HDGT had a higher use of medication and those in the LaGT had a higher use of antidepressants compared to the LGT.

### Comparison with existing literature

Bereavement has consistently been associated with increased use of health care and psychotropic medication (4, 5, 18). The present study establishes that individuals most likely to receive excess health care services beyond 3 years after bereavement were those with high levels of persistent grief symptoms. The long-term findings regarding health care are likely to be impacted by events such as illness of the relative or other losses during the ten-year period after the patient's death. However, such events may also impact the other grief trajectory groups.

A prior study of health care use before bereavement showed that the HGT was associated with more contacts to GP, medication use, and use of mental health services (24). The relatives in the HGT may have predisposing vulnerabilities and challenges and that severe illness and bereavement pose a high level of distress that they find difficult to handle. Furthermore, relatives with HGT had a lower educational level for both spouses and adult children of the ill person (23). Hence, prior mental health conditions and low educational level are risk factors for long-term psychological distress in bereaved relatives. Nevertheless, the findings of the present study may be connected with confounding by indication as the persons in the HGT had higher health care use prior to bereavement, but the findings underline that relatives in the HGT are persons who are likely to need additional support from the health care system.

The contact pattern to out-of-hours GP services was diverging. These services are intended for acute situations and available free of charge to all citizens in Denmark (37). Hence, it is at first sight encouraging that persistent grief symptoms may not significantly affect the long-term use of these services as this could indicate that the supportive services provided during daytime sufficiently support the relatives. However, the connection between daytime consultation and out-of-hours use may be complex and those with need for mental health support might call helplines for mental health. A recent study showed that the use of out-of-hours GP services among citizens above 75 years of age was not lower if the patient had more daytime consultations in general practice (38), and personal characteristics rather than organizational factors were suggested to affect the use of out-of-hours GP services. Hence, out-of-hours contact may not be relevant health care for the participants.

Regarding psychotropic medication, the use of antidepressant and anxiolytics and sedatives was significantly higher in the HDGT and HGT than in the LGT. Approximately 18% used psychotropic medication in the first year after bereavement (39). Our results indicate that early grief patterns may predict health care use is a new finding that extends prior knowledge of higher medication use in bereaved compared to non-bereaved (4, 18). Hence, a specific group of relatives may be recognizable for intervention, especially as the HGT has been found to be associated with higher medication use already before bereavement (24).

We found that relatives with HGT had a higher use of any mental health service after bereavement. Hence, the available services seem to be directed at those with the most severe symptoms. However, the persistent need for mental health service over a long-term period could indicate that the existing interventions may not be sufficient to address the needs of bereaved relatives. Also, the vulnerability of the relatives in the HGT group may also be related to prior health and mental health conditions contributing to their need for individually adapted mental health services.

Talk therapy sessions with the GP and private-practice psychologists early in the bereavement period have been found to reduce the risk of suicide, deliberate self-harm, and reduce psychiatric hospitalization in bereaved persons (40). The HGT was associated with GP talk therapy but not psychotherapy by a psychologist among relatives *before* the death (24). Thus, general practice holds an important task to support those in the HGT, who are more likely to have a low educational level and may not have the economic or mental resources to attend therapy with a psychologist. In recent years, more attention has been drawn at relatives and new knowledge has emerged to inform health professionals, including a supportive intervention in general practice using a dialogue questionnaire (41) and the integrated process model of loss and grief to support the understanding of grief in general (1).

We found that relatives with HGT had a higher mortality compared to relatives with LGT. Previous studies did not compare mortality according to symptoms levels, but excess mortality has been found for bereaved compared to non-bereaved persons in most (3, 17), but not all previous studies (18). Bereaved relatives constitute a heterogeneous group with different risk factors for experiencing adverse bereavement outcomes (3). Caregiving for a close relative have been suggested to buffer the stress reaction in relatives (19). However, if the relative suffers from distress due to their own physical illness this may add to their vulnerability and grief symptoms. Development of persistent high grief symptoms may be due to several risk factors, and our findings support that comorbidity is likely to play an important role as an intrapersonal risk factor for bereavement outcome (12).

### Strengths and limitations

The main advantages of the current study are the follow-up time of 10 years, the longitudinal study design of grief symptom assessment at three time points in 1,735 relatives, and the combination with valid register data on health care services. The Danish registers are considered almost complete, and the data can be linked precisely (42). We used grief trajectories based on repeated grief measurements as exposure, which can be considered a strength as persistent grief reactions were included. The association between having persistent high levels of grief symptoms and higher use of health care services may be considered to be confounding by indication as those in the exposure group HGT with the highest symptom level may have an a priori higher use of health care due to symptoms.

The study was limited by the number of participants and thus lacked power to investigate for instance the different types of mental health services. Also, the follow-up of 10 years may be seen as a limitation to reveal long-term impacts of bereavement on mortality. The study included no comparison group from the background population, which was a limitation. Beyond seven years of bereavement, the association of higher use of primary health care in the HGT seemed to level out. This may indicate that the impact of bereavement diminishes over time. However, it could also be due to the group size as a small group of relatives in the HGT could potentially have had many contacts and this effect would diminish if they had died during follow-up.

The study population was younger and had a higher educational level than non-participants (18, 23). As those with high education may have lower symptom levels, the prevalence of high grief symptoms might have been underestimated in the present study. Still, we believe that the results may be generalizable to bereaved relatives in Denmark and in countries with similar health care systems.

## Clinical implications

Despite increased use of health care, including mental health services, those in the HGT had a persistently high level of symptoms. Also, the higher use of medication in the group may indicate a need for further intervention. Bereavement support has been shown to benefit symptomatic persons with psychological distress and need of intervention (43). These findings highlight the importance of identifying those at risk as it may allow for timely intervention, including referral to extended mental health services. For instance, the relatives' symptom levels and needs for support could be systematically assessed by health professionals in primary and secondary care, preferably already before the patient's death.

Individuals with elevated grief symptoms were more likely to engage with general practice in the present study. In contrast, a previous study in the US found that relatives with a high grief symptom level underutilized healthcare measured as contacts to the health care system (44). These differences suggest that in settings with universal healthcare access, the opportunity for contact with vulnerable relatives may be better. Hence, this enables health professionals to proactively plan a follow-up appointment regarding mental health symptoms.

## Future research

To mitigate adverse bereavement reactions, further studies are needed to explore the effects and implementation of targeted intervention in primary care. Such studies should focus on individuals at risk of complications or showing symptoms of distress, such as intensive levels of grief (43). Supportive interventions in general practice starting before bereavement needs further implementation (45), and online interventions show promising results for further implementation to address the increasing demand for mental health support (46). Additionally, we need to further explore the pathways of shared care between health care sectors and the availability of mental health services for bereaved relatives in primary and secondary care and improve collaboration and implementation of evidence-based interventions.

## Conclusion

In this follow-up study, we found that relatives with persistently high grief symptoms had more frequent contact with primary care up to seven years after bereavement and higher use of psychotropic medication, mental health services and mortality for at least 10 years after bereavement.

These results extend our earlier findings, showing that relatives in the HGT are vulnerable and already have higher primary care use before the patient's death. Moreover, despite seeking mental health care, these relatives continue to use more medication. Thus, the existing interventions may not be sufficient since this group seems to need long-term support. The present findings highlight the need for targeted interventions of long-term support, particularly in primary care, to adequately address the needs of this high-risk group of bereaved relatives.

## Data Availability

The datasets presented in this article are not readily available because of GDPR. Requests to access the datasets should be directed to the first author.
