# Correction: Grief trajectories and long-term health effects in bereaved relatives: a prospective, population-based cohort study with ten-year follow-up

**DOI:** 10.3389/fpubh.2025.1678380

**Published:** 2025-10-30

**Authors:** Mette Kjærgaard Nielsen, Kaj Sparle Christensen, Mette Asbjoern Neergaard, Pernille Envold Bidstrup, Mai-Britt Guldin

**Affiliations:** ^1^Department of Mental Health, Research Unit for General Practice, Aarhus, Denmark; ^2^Department of Public Health, Aarhus University, Aarhus, Denmark; ^3^Section for Specialist Palliative Care, Department of Oncology, Aarhus University Hospital, Aarhus, Denmark; ^4^Department of Clinical Medicine, Aarhus University, Aarhus, Denmark; ^5^Psychological Aspects of Cancer, Danish Cancer Institute, Copenhagen, Denmark

**Keywords:** grief, bereavement, relatives, general practice (GP), health care use, mortality, medication use, primary care

Author Henrik Schou Pedersen was erroneously included as an author in the published article. The correct author list reads: **Mette Kjærgaard Nielsen, Kaj Sparle Christensen, Mette Asbjoern Neergaard, Pernille Envold Bidstrup, and Mai-Britt Guldin**.

The Author Contributions Statement has been corrected to read:

“MKN: Investigation, Writing – review & editing, Conceptualization, Methodology, Resources, Funding acquisition, Visualization, Project administration, Validation, Formal analysis, Data curation, Writing – original draft. KS: Writing – review & editing, Funding acquisition, Supervision, Conceptualization, Project administration, Validation, Methodology. MAN: Project administration, Funding acquisition, Writing – review & editing, Methodology, Formal analysis, Validation, Resources, Visualization, Investigation, Conceptualization, Supervision, Data curation. PB: Visualization, Formal analysis, Resources, Data curation, Validation, Methodology, Supervision, Investigation, Conceptualization, Funding acquisition, Writing – review & editing. M-BG: Formal analysis, Writing – review & editing, Supervision, Methodology, Investigation, Visualization, Data curation, Conceptualization, Validation, Funding acquisition, Resources.”

The acknowledgments has been updated to acknowledge Henrik Schou Pedersen. The corrected Acknowledgments appears below:

“We wish to extend our profound gratitude to patients and relatives who participated in this study. Thanks to the staff at the Research Unit for General Practice, Aarhus, Denmark, in particular Henrik Schou Pedersen for data management and choice of statistical analyses, data manager Kaare Rud Flarup, and language editor Lone Niedziella. Furthermore, the authors thank statistician Anders Helles Carlsen, Aarhus University Hospital—Psychiatry, Denmark, for his work on grief trajectories.”

In the published article, there were errors in the notation of the odds ratios and hazard ratios.

In the **Abstract**, paragraph Results, the sentence previously read:

“The HGT was associated with higher use of mental health services [OR = 2.86 (95% CI 1.58; 5.19)], antidepressants [OR = 5.63 (95% CI 3.52; 9.01)], sedatives and anxiolytics [OR = 2.60 (95%CI 1.63; 4.14)], and excess mortality [OR = 1.88 (95% CI 1.1; 3.2)] compared to the LGT.”

The corrected sentence appears below:

“The HGT was associated with higher use of mental health services [OR = 2.86 (95% CI 1.58; 5.19)], antidepressants [OR = 5.63 (95% CI 3.52; 9.01)], sedatives and anxiolytics [OR = 2.60 (95% CI 1.63; 4.14)], and excess mortality [HR = 1.88 (95% CI 1.1; 3.2)] compared to the LGT.”

A correction has been made to the section **Materials and methods**, *Statistical analysis*, paragraph 5.

The sentence previously read:

“All estimates were presented with 95% confidence intervals (CIs) and ORs were considered statistically significant if 1 was not included in the CI.”

This has been corrected to read:

“All estimates were presented with 95% confidence intervals (CIs) and ratio estimates were considered statistically significant if 1 was not included in the CI.”

A correction has been made to the section **Results**, *Mortality*, paragraph 1.

The sentence previously read:

“In total, 186 (10.7%) died during 3–10 years after the patient's death, and the HGT was associated with mortality [OR = 1.88(95%CI: 1.11;3.21)] compared to the LGT (Table 3).”

This has been corrected to read:

“In total, 186 (10.7%) died during 3–10 years after the patient's death, and the HGT was associated with mortality [HR = 1.88 (95% CI: 1.11; 3.21)] compared to the LGT (Table 3).”

The original article has been updated.

